# Inverse Relationship between Apolipoprotein A-I and Cerebral White Matter Lesions: A Cross-Sectional Study in Middle-Aged and Elderly Subjects

**DOI:** 10.1371/journal.pone.0097113

**Published:** 2014-05-12

**Authors:** Ze-Gang Yin, Ling Li, Min Cui, Shi-Ming Zhou, Ming-Ming Yu, Hua-Dong Zhou

**Affiliations:** 1 Department of Neurology, Daping Hospital and Institute of Field Surgery, Third Military Medical University, Chongqing, China; 2 Department of Neurology, Wuhan General Hospital of PLA, Wuhan, Hubei, China; INRCA, Italy

## Abstract

**Background:**

Apolipoprotein A-I (apoA-I), the major protein for high density lipoprotein, is essential for reverse cholesterol transport. Decreased serum levels of apoA-I have been reported to correlate with subcortical infarction and dementia, both of which are highly related to white matter lesions (WMLs). However, the association between apoA-I and WMLs has never been investigated. In this study, we sought to investigate the association between apoA-I and the presence of WMLs in middle-aged and elderly subjects.

**Methods:**

Consecutive patients aged 50 years and older of our department were prospectively enrolled in this study (n = 1282, 606 men and 676 women, 65.9±9.4 years). All participants underwent MRI scans to assess the presence and severity of WMLs. Multivariate logistic regression analyses were performed to examine the association of apoA-I with WMLs.

**Results:**

Patients with WMLs were older and showed significantly higher proportion of male sex, hypertension, diabetes mellitus, previous stroke, and coronary heart disease whereas levels of total cholesterol, high density lipoprotein cholesterol, and apoA-I were lower. After adjustment for potential confounders, the lowest apoA-I quartile was independently associated with an increased risk of WMLs (odds ratio: 1.87, 95% confidence interval: 1.29–2.72). In sex-specific analyses, this relationship was observed only in women.

**Conclusions:**

Our findings demonstrated that apoA-I was inversely associated with the presence of WMLs in middle-aged and elderly subjects. This results suggest that therapies which increase apoA-I concentration may be beneficial to reduce the risk of WMLs, dementia and stroke.

## Introduction

Cerebral white matter lesions (WMLs), also known as leukoaraiosis, are frequently observed on brain magnetic resonance imaging (MRI) in older individuals. Accumulating evidence suggests that WMLs have substantial clinical impact through associations with dementia, disability, depression, stroke, and mortality [1], [2]. WMLs reflect multiple pathologic changes, including loss and deformation of myelin sheath, changes in vessel wall permeability, disruption of the blood-brain barrier, hypoperfusion attributable to altered cerebrovascular autoregulation, fluid shift from the ventricles and gliosis [3], [4]. Whereas advanced age and hypertension are the most widely accepted risk factors for WMLs, the current understanding of other risk factors for WMLs remains less clear. The role of lipids in the pathogenesis of WMLs is controversial in former studies. Some studies have shown that low levels of high density lipoprotein (HDL) cholesterol and hypertriglyceridemia may increase the risk of WMLs [5], [6]. However, other authors have not consistently shown similar associations [7], [8].

Apolipoprotein A-I (apoA-I) is the major protein component of HDL and plays an important role in transporting excess cholesterol from peripheral cells to the liver [9]. Besides the atheroprotective effect, apoA-I also manifests anti-inflammatory and antioxidant effects [10]. Recently, decreased serum apoA-I levels have been reported to increase the risk of deep subcortical infarction [11], which often coexists with WMLs in brains [12]. Furthermore, the Honolulu-Asia aging study has found an inverse relation between apoA-I and dementia [13]. However, the association between apoA-I and the presence of WMLs has never been investigated.

Thus, in the present cross-sectional study, we aimed to evaluate the association of apoA-I with the presence of WMLs in middle-aged and elderly subjects. This could improve our understanding of the pathophysiology underlying this highly prevalent cerebrovascular disease.

## Methods

### Ethics Statement

The study protocols were approved by Institutional Review Board of the Third Military Medical University and performed in accordance with the Declaration of Helsinki. All participants provided written informed consent prior to their inclusion in the study.

### Study population

Consecutive inpatients admitted to the department of neurology of Daping Hospital in the city of Chongqing from June 2012 to June 2013 were prospectively enrolled in this study. All patients were proposed to undergo brain MRI scans. Inclusion criteria were (1) aged 50 years and older; (2) ability to understand the aim of the study and provide written informed consent. The following patients were excluded: (1) patients with leukoencephalopathy of nonvascular origin (immunological-demyelinating, toxic, infectious, other); (2) patients with brain tumors, dementia, psychoses; (3) patients who had contraindications for MRI scans or refused to undergo cerebral MRI; (4) patients who had been using lipid-lowering medications before admission; (5) patients who refused to undergo lipids tests.

### Clinical information collection

Every participant underwent a standardized clinical examination and interview using a detailed questionnaire survey to obtain information including demographic data, past medical history, current cigarette smoking status, and the use of antihypertensive medications, lipid-lowering medications, and oral hypoglycemic agents or insulin. Body mass index was calculated as the weight divided by the square of the height (kg/m^2^). Blood pressure was determined using an aneroid sphygmomanometer with the patients in a sitting position after relaxing for at least 10 minutes, and the mean of two measurements was used. Blood samples were drawn in the morning after an overnight fast and sent to the clinical laboratory of Daping Hospital for the measurement of serum indices. The levels of fasting blood glucose (FBG), total cholesterol (TC), triglyceride, HDL cholesterol, and low density lipoprotein cholesterol were measured by standard enzymatic techniques. The levels of apoA-I and apoB were measured by the immunoturbidimetric method using a DxC800 chemistry analyzer (Beckman Coulter Inc., Brea, California, United States). The intra- and inter-assay coefficient of variation for apoA-I were 1.9% and 2.3%, respectively. Hypertension was defined as systolic/diastolic blood pressure measures greater than 140/90 mmHg, or current treatment with antihypertensive medications. Diabetes mellitus was defined as FBG ≥7.0 mmol/L or current treatment with hypoglycemic agents or insulin. In addition, a physician's diagnostic report of cardiovascular disease, including coronary heart disease (CHD) or previous stroke, was gathered for each participant.

### MRI scans and WMLs grading

MRI was performed following a standard protocol including T1- (TR/TE: 450/8.9 ms) and T2- (TR/TE: 5000/87 ms) weighted and fluid attenuated inversion recovery (FLAIR, TR/TE: 8500/88 ms, inversion time: 2000 ms) sequences using a 1.5 T magnet (Signa EXCITE HD 1.5T, General Electric, USA). The degree of WMLs severity was rated on FLAIR by two trained investigators (Yin and Cui) who were blind to the clinical data, using the modified visual scale of Fazekas et al [14]. Disagreements of imaging analysis were resolved by consensus. Taking into account only deep and subcortical white matter, lesions were classified into three categories: mild  =  single lesions must be more than 3 mm and smaller than 10 mm, areas of grouped lesions must be smaller than 20 mm in any diameter; moderate  =  single lesions between 10 and 20 mm, areas of grouped lesions more than 20 mm in any diameter, no more than connecting bridges between individual lesions; severe  =  single lesions or confluent areas of hyperintensity 20 mm or more in any diameter [15].

### Statistical analyses

Demographic data were expressed in percentages for categorical variables and compared using the chi-square test. Continuous variables were expressed as mean ± SD and compared with a Student *t* test for factors with a normal distribution or expressed as median and interquartile range and compared with the Mann-Whitney *U* test for factors that were not normally distributed. The relationship between apoA-I quartiles and severity of WMLs was evaluated by chi-square linear-by-linear association test. Multivariate logistic regression analyses were performed to determine whether the decreased serum apoA-I levels were independently associated with WMLs after adjustment for the potential confounders. The baseline variables having *p*<0.10 for the presence of WMLs in univariate analyses were selected to enter the multivariate models. Finally, we repeated all analyses excluding participants within the acute period of stroke (n = 220). Odds ratios (ORs) with 95% confidence intervals (CIs) were calculated. All *p* values were two-tailed, and values of *p*<0.05 were considered statistically significant. All statistical analyses were performed using SPSS18.0 for Windows (SPSS Inc., Chicago IL).

## Results

1509 consecutive patients aged 50 years and older were admitted to the department of neurology of Daping Hospital from June 2012 to June 2013. We excluded 101 individuals who had contraindications for MRI scans or refused to undergo brain MRI, 14 who were with leukoencephalopathy of nonvascular origin, 35 who were diagnosed with brain tumors, dementia, or psychoses, 45 who had been using lipid-lowering medications before admission, and 32 who refused to undergo lipids tests. Finally, a total of 1282 patients were enrolled in the study. Among the 1282 patients, 587 complained of non-specific neurological symptoms (e.g., dizziness, vertigo, numbness and other symptoms), 220 of acute stroke, 102 of headache, 81 of sleep disorders, 65 of movement disorders, 125 of peripheral neuropathy and 102 of other neurological conditions.

The mean age of the study population was 65.9±9.4 years; 47.3% were men. Among the 1282 participants, mild WMLs was found in 486 (37.9%), moderate WMLs in 147 (11.5%), and severe WMLs in 91 (7.1%). Demographic characteristics of the study population are shown in Table 1. Patients with WMLs were older (*p*<0.001) and more likely to be male (*p*<0.001) in comparison with those without WMLs. Moreover, patients with WMLs showed significantly higher proportion of hypertension, diabetes mellitus, previous stroke, and CHD whereas levels of TC, HDL cholesterol, and apoA-I were lower.

**Table 1:** Clinical characteristics of subjects with or without cerebral white matter lesions.

Clinical characteristics^a^	All subjects (n = 1282)	Subjects without WMLs (n = 558)	Subjects with WMLs (n = 724)	P value
Age (years)	65.9±9.4	61.6±7.7	69.2±9.1	<0.001
Men (%)	606 (47.3)	231 (41.4)	375 (51.8)	<0.001
Current smoking (%)	223 (17.4)	103 (18.5)	120 (16.6)	0.378
Previous stroke (%)	158 (12.3)	23 (4.1)	135 (18.6)	<0.001
Coronary heart disease (%)	177 (13.8)	43 (7.7)	134 (18.5)	<0.001
Hypertension (%)	621 (48.4)	176 (31.5)	445 (61.5)	<0.001
Diabetes (%)	228 (17.8)	83 (14.9)	145 (20.0)	0.017
Use of antihypertensive agents (%)	466 (36.3)	125 (22.4)	341 (47.1)	<0.001
Use of anti-diabetic agents (%)	145 (11.3)	52 (9.3)	93 (12.8)	0.048
Body mass index (kg/m^2^)	23.6±3.4	23.4±3.3	23.7±3.5	0.205
Systolic blood pressure (mmHg)	135.4±17.2	131.6±16.1	138.4±17.5	<0.001
Diastolic blood pressure (mmHg)	81.7±10.1	80.5±9.8	82.6±10.3	<0.001
Fasting plasma glucose (mmol/L)	5.05 (4.60–5.78)	5.01 (4.61–5.63)	5.05 (4.59–5.89)	0.386
TC (mmol/L)	4.89±1.04	5.01±1.04	4.80±1.04	<0.001
Triglyceride (mmol/L)	1.23 (0.89–1.76)	1.27 (0.89–1.81)	1.21(0.90–1.74)	0.208
HDL cholesterol (mmol/L)	1.21±0.33	1.26±0.34	1.17±0.32	<0.001
LDL cholesterol (mmol/L)	2.67±0.73	2.71±0.74	2.64±0.73	0.105
ApoA-I (g/L)	1.38±0.25	1.43±0.25	1.34±0.25	<0.001
ApoB (g/L)	0.92±0.22	0.93±0.23	0.91±0.22	0.150

Abbreviation: WMLs, white matter lesions; TC, total cholesterol; HDL, high density lipoprotein; LDL, low density lipoprotein; ApoA-I, apolipoprotein A-I; ApoB, apolipoprotein B.

a Results are expressed as mean ± SD for variables with a normal distribution, as median and interquartile range for variables with a skewed distribution, or as number with percentage for categorical variables.

**Figure 1:**
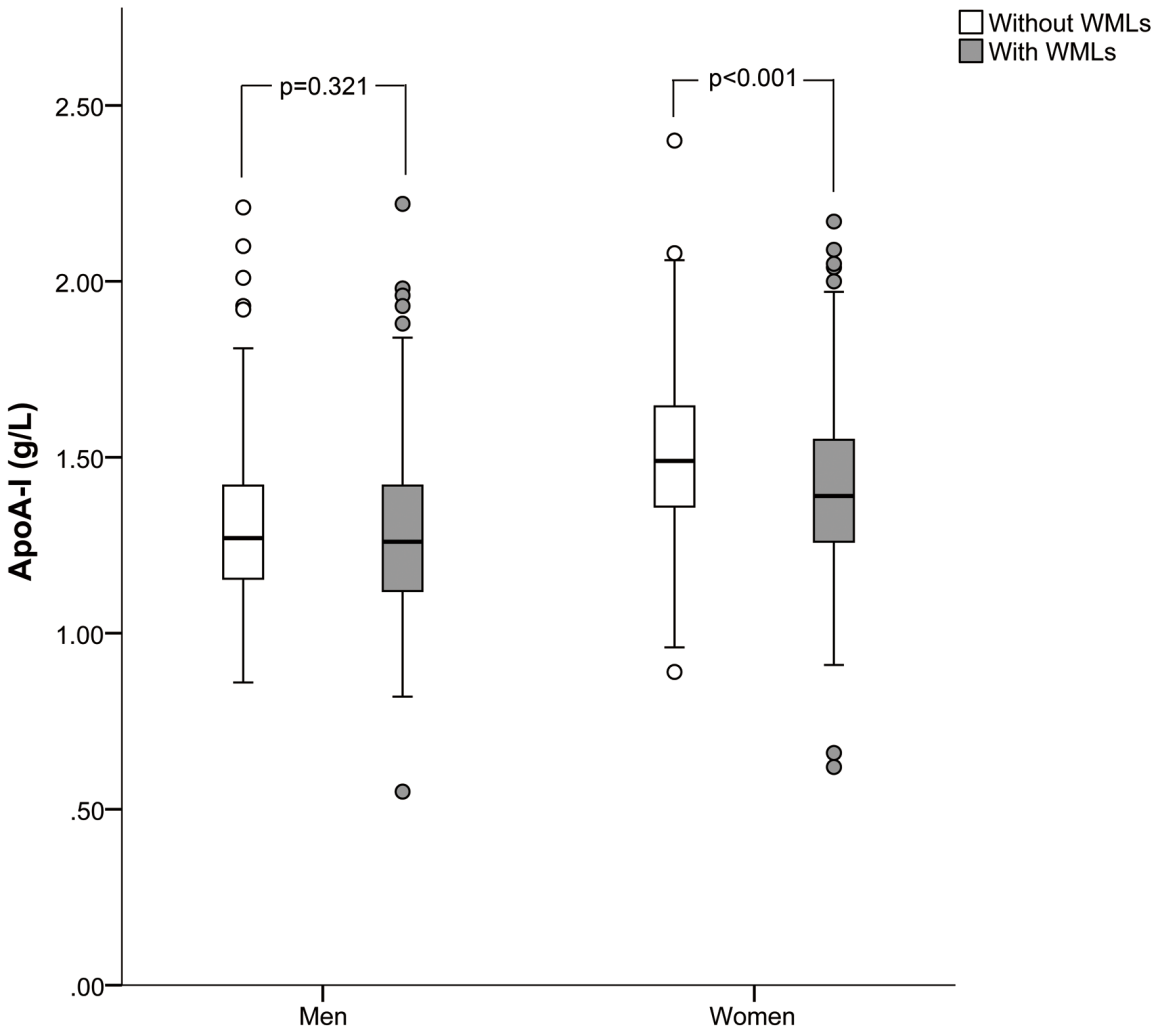
Box-plots of apoA-I values and their association WMLs in men and women. ApoA-I, apolipoprotein A-I; WMLs: white matter lesions.

**Figure 2:**
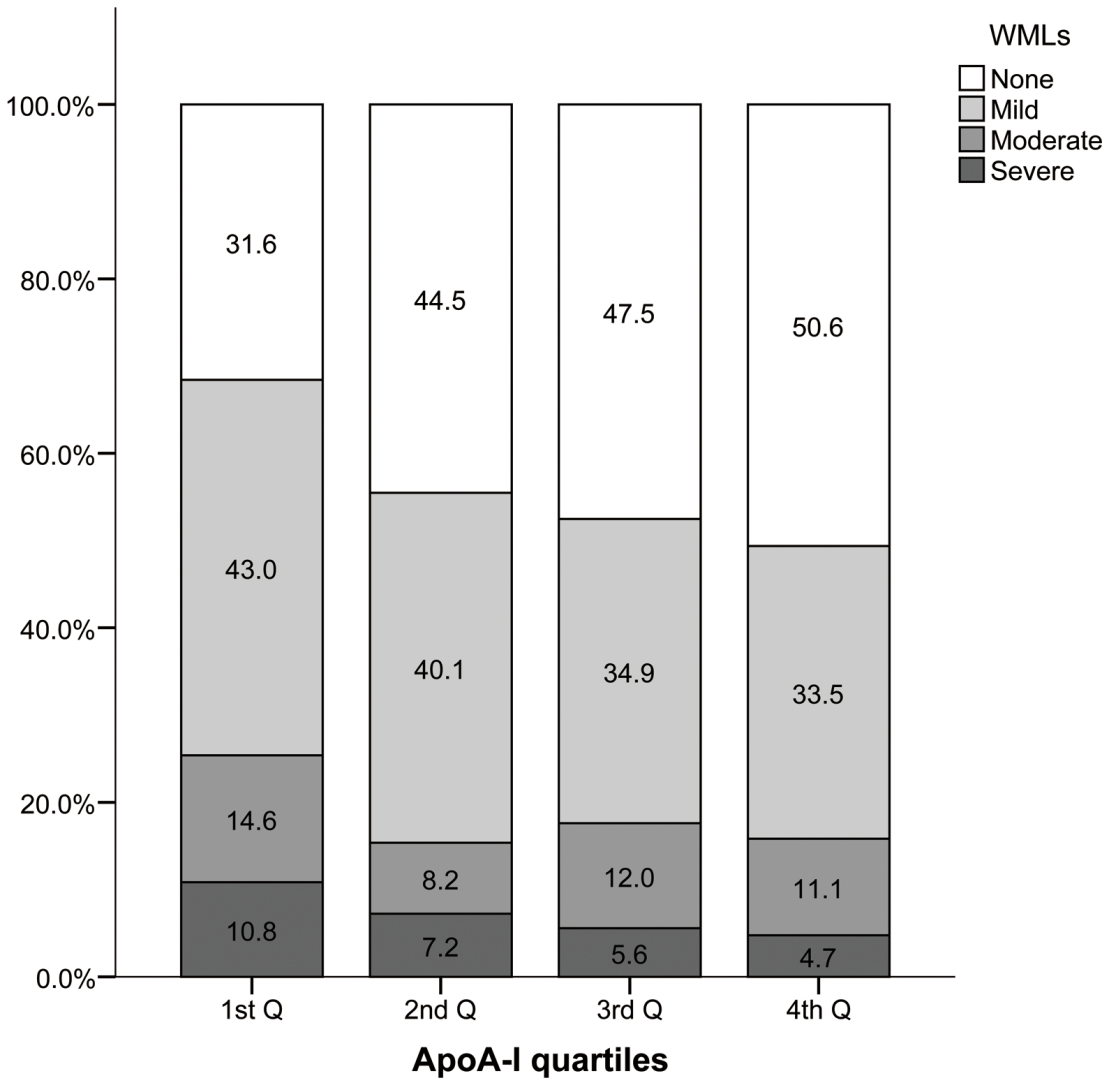
Presence and severity of WMLs according to apoA-I quartiles. Values are percentages of patients. As levels of apoA-I increased, the presence and severity of WMLs increased (*P* for trend <0.001). ApoA-I, apolipoprotein A-I; WMLs: white matter lesions.

Serum apoA-I levels ranged from 0.62 to 2.40 g/L for women and 0.47 to 2.22 g/L for men. They were higher for women than men (1.46±0.24 vs. 1.29±0.23 g/L; *p*<0.001). As shown in Figure 1, in women, those with WMLs had lower levels of apoA-I than those without WMLs (1.40±0.24 vs. 1.51±0.23 g/L; *p*<0.001). However, this difference was not observed between the two groups in men (1.28±0.24 vs. 1.30±0.22 g/L; *p* = 0.321). Patients were stratified into quartiles according to the serum apoA-I levels by sex. The sex-specific quartiles were ≤1.30, 1.31 to 1.46, 1.47 to 1.60, and ≥1.61 g/L for women while ≤1.13, 1.14 to 1.26, 1.27 to 1.42, and ≥1.43 g/L for men. As shown in Figure 2, patients with lower apoA-I quartiles were likely to have higher prevalence and severity of WMLs (*p* for trend <0.001).

Table 2 shows the results of the multivariate logistic regression model and the OR for each factor. After adjustment for age, sex, history of stroke, CHD, hypertension, and diabetes, patients with the lowest apoA-I quartile were approximately 1.9 times more likely to have WMLs, compared with those with the highest apoA-I quartile. When the apoA-I concentration entered the model as a continuous variable, this inverse relationship between apoA-I and WMLs remained significant (OR: 0.40, 95% CI: 0.23–0.70). In addition, age, previous stroke and hypertension were independently associated with an increased risk of WMLs (*p*<0.001 for all variables). In sex-specific analyses, the lowest apoA-I quartile still predicted higher prevalence of WMLs in women (OR: 3.46, 95% CI: 2.08–5.76), but not in men (Table 3). When the apoA-I concentration was added to the multivariate models as a continuous variable, the sex difference still existed (OR: 0.17, 95%CI: 0.08–0.36 for women; OR: 1.20, 95%CI: 0.52–2.77 for men).

**Table 2:** Multivariate analyses of white matter lesions determinants.

Variables	OR (95% CI)	P value
Age	1.10 (1.08–1.11)	<0.001
Sex, male	1.28 (0.99–1.65)	0.059
Previous stroke	2.74 (1.67–4.52)	<0.001
Coronary heart disease	1.22 (0.80–1.87)	0.359
Hypertension	2.47 (1.89–3.23)	<0.001
Diabetes	0.78 (0.54–1.11)	0.166
ApoA-I quartiles^a^		
First	1.87 (1.29–2.72)	0.001
Second	1.22 (0.85–1.73)	0.279
Third	1.11 (0.78–1.58)	0.568
Fourth	Reference	
P for trend		0.001

Abbreviation: ApoA-I, apolipoprotein A-I.

a Ranges for sex-specific quartiles were ≤1.30, 1.31 to 1.46, 1.47 to 1.60, and ≥1.61 g/L for women while ≤1.13, 1.14 to 1.26, 1.27 to 1.42, and ≥1.43 g/L for men.

**Table 3:** Multivariate analyses of white matter lesions determinants stratified by sex.

Variables	Men (n = 606)		Women (n = 676)	
	OR (95% CI)	P value	OR (95% CI)	P value
Age	1.09 (1.07–1.12)	<0.001	1.11 (1.08–1.13)	<0.001
Previous stroke	2.35 (1.27–4.37)	0.007	3.94 (1.65–9.42)	0.002
Coronary heart disease	1.22 (0.67–2.25)	0.519	1.29 (0.70–2.35)	0.413
Hypertension	3.20 (2.15–4.75)	<0.001	1.98 (1.36–2.89)	<0.001
Diabetes	0.82 (0.49–1.36)	0.442	0.76 (0.45–1.29)	0.311
ApoA-I quartiles^a^				
First	0.86 (0.49–1.51)	0.605	3.46 (2.08–5.76)	<0.001
Second	0.85 (0.50–1.44)	0.546	1.61 (0.99–2.64)	0.058
Third	0.91 (0.54–1.53)	0.714	1.31 (0.80–2.15)	0.288
Fourth	Reference		Reference	
P for trend		0.561		<0.001

Abbreviation: ApoA-I, apolipoprotein A-I.

a Ranges for sex-specific quartiles were ≤1.30, 1.31 to 1.46, 1.47 to 1.60, and ≥1.61 g/L for women while ≤1.13, 1.14 to 1.26, 1.27 to 1.42, and ≥1.43 g/L for men.

Furthermore, we repeated the multivariate adjusted logistic regression analyses after excluding 220 patients within the acute period of stroke, and found that the lowest apoA-I quartile remained an independent predictor for WMLs (OR: 2.13, 95% CI: 1.42–3.20). In addition, the effects of HDL cholesterol on WMLs were similar to those of apoA-I in multivariate logistic regression analyses (data not show). The Akaike information criterion (AIC) was used to compared the goodness of fit between the two models (one including apoA-I and the other including HDL cholesterol). As a result, the model with apoA-I showed a slightly better fit than the model using the HDL cholesterol levels (1441 vs. 1447 of the AIC value, respectively).

## Discussion

In the current study carried out on middle-aged and elderly subjects, we investigated the associations of lipid profiles and apolipoproteins with the presence of WMLs, and found that patients with the lowest apoA-I quartile had approximately a 1.9 times increased risk for WMLs. This association was independent of age, sex, and vascular risk factors. Further analyses showed that this relationship existed only in women. These findings did not change after excluding 220 patients within the acute period of stroke. To the best of our knowledge, we first demonstrated that apoA-I was inversely associated with an increased risk of WMLs. This might provide a partial explanation of the previously observed association between decreased serum levels of apoA-I and dementia in elderly adults [13], [16].

Dyslipidemias are widely recognized as a risk factor for stroke [17], [18], and lipid-lowering therapies have demonstrated benefits in stroke prevention and prognosis [19], [20]. However, the association between serum lipids and WMLs remains inconsistent in former studies. For instance, Crisby et al. [6] reported that HDL cholesterol was inversely related to the risk of WMLs, in accord with our study. In contrast, a study investigating the association between metabolic syndrome components and WMLs found that hypertriglyceridemia, but not low level of HDL cholesterol, was an independent risk factor for WMLs [21]. Likewise, hypertriglyceridemia was significantly related to severe WMLs in a recent study [5]. This relationship, however, was not observed in our study. Furthermore, TC was an independent risk factor for WMLs in some studies [22], [23], but was protective against WMLs in two cohorts [24]. We found that subjects without WMLs had higher levels of TC, which suggested a protective role of TC against WMLs. However, this relationship between TC and WMLs was no longer significant after adjustment for potential confounding variables.

ApoA-I is essential for reverse transport of cholesterol from peripheral tissue to the liver [9]. It also has antioxidant and anti-inflammatory effects [10]. In contrast, apolipoprotein B (apoB) is a major structural protein for very low density-low density lipoprotein spectrum and reflects atherogenic potential [9]. The apoB/apoA-I ratio is increasingly recognized as a better predictor of cardiovascular disease than other traditional cholesterol measures [25], [26]. Very few reports from the literature are available to compare with our findings regarding apolipoproteins and WMLs. Cross-sectional data from the community-dwelling Austrian Stroke Prevention Study demonstrated that participants with microangiopathy-related cerebral damage had lower levels of apoA-I. However, decreased apoA-I concentration did not enter the final multivariate model [27]. In the present study, we observed that patients with WMLs had lower levels of apoA-I, compared with those without WMLs. The apoB levels, however, did not show significant difference between the two groups. These findings are consistent with a recent study, which reported that levels of apoA-I, but not apoB, were associated with deep subcortical infarction [11]. These results indicate that the effects of apolipoproteins on cerebral small vessel disease might differ from those on large vessel disease. Anti-inflammatory effects of apoA-I may play a more important role in preventing WMLs formation, than atheroprotective effects.

Although the exact mechanism underlying the inverse relationship between apoA-I and WMLs remains to be elucidated, there are some plausible explanations. First, the interaction of apoA-I with ATP-binding cassette transporter A1 (ABCA1) activates signal transducer and activator of transcription 3, which suppresses the production of inflammatory cytokines and ultimately inhibits the inflammatory response [28]. Biomarkers of inflammation such as interleukin-6, intercellular adhesion molecule, and C-reactive protein have been reported to be associated with the presence or progression of WMLs in population-based studies [29]–[31]. In animal model, neuroinflammation has also proved to be an important mechanism of white matter damage [32]. Second, apoA-I binds amyloid β (Aβ) and prevents Aβ-induced neurotoxicity [33], and apoA-I deficiency increases levels of deposited Aβ in the brain vessels [34]. These results suggest that apoA-I attenuates cerebral amyloid angiopathy, which is not only a hallmark of Alzheimer's disease [12], but also a predictor for WMLs [35], [36]. Third, apoA-I promotes reverse cholesterol transport through the macrophage ABCA1 and protects large vessels from atherosclerosis [37], which has been related to WMLs in several studies [38], [39]. Furthermore, apoA-I may directly protect small vessels from microatheroma which is a manifestation of arteriolosclerosis, the primary pathological feature of small vessel disease [12].

Additionally, we found that the lowest apoA-I quartile was associated with WMLs only in women. Similarly, a longitudinal study has found that higher HDL cholesterol predicted better maintenance of cognitive abilities in women, but not in men [40]. The mechanisms underlying the sex differences are unknown. We cannot rule out a possible effect modification of sex hormones in the association. Sex differences should be considered in future studies of the effect of lipids on cerebral small vessel disease.

The present study has several limitations. First, the present study was cross-sectional and could not determine the exact causality between decreased serum apoA-I concentration and WMLs. Second, the study population was composed of hospital-based patients and appeared to have more vascular risk factors than community-based cohorts, which would restrict the generalization of our results.

In summary, we demonstrated that serum apoA-I levels were inversely associated with the presence of WMLs in middle-aged and elderly subjects. This association was observed only in women. Apart from HDL cholesterol, traditional serum lipid levels were not independently associated with WMLs. Although further longitudinal studies are needed to confirm the conclusions and to elucidate the mechanisms for the association, our results suggest that therapies which increase apoA-I concentration may be beneficial to reduce the risk of WMLs, dementia and stroke.
